# Racial and ethnic disparities in cancer caregiver burden and potential sociocultural mediators

**DOI:** 10.1007/s00520-022-07367-x

**Published:** 2022-10-03

**Authors:** Anny T. H. R. Fenton, Katherine A. Ornstein, Peggye Dilworth-Anderson, Nancy L. Keating, Erin E. Kent, Kristin Litzelman, Andrea C. Enzinger, Julia H. Rowland, Alexi A. Wright

**Affiliations:** 1 Dana-Farber Cancer Institute, Boston, MA USA; 2 John Hopkins University, School of Nursing, Baltimore, MD USA; 3 Department of Health Policy and Management, Gillings School of Global Public Health, University of North Carolina, Chapel Hill, NC USA; 4 Harvard Medical School, Boston, MA USA; 5 Brigham and Women’s Hospital, Boston, MA USA; 6 Lineberger Comprehensive Cancer Center, University of North Carolina, Chapel Hill, NC USA; 7 University of Wisconsin–Madison, Madison, WI USA; 8 University of Wisconsin Carbone Cancer Center, Madison, WI USA; 9 Smith Center for Healing and the Arts, Washington, DC USA

**Keywords:** Racial and ethnic disparities, Caregiving, Caregiving burden, Social support, Caregiving preparedness

## Abstract

**Purpose:**

Black and Hispanic cancer patients experience many worse care quality and health outcomes than non-Hispanic White patients, yet less is known about disparities in caregiving responsibilities and burden among cancer caregivers.

**Methods:**

We analyzed cross-sectional data from Cancer Care Outcomes Research and Surveillance consortium, a large multi-regional, population-based study of colorectal and lung cancer patients and their caregivers. Bivariate and multivariable regression models assessed differences by racial and ethnic groups in caregiving responsibilities and social/emotional, financial, and health burdens. Structural equation models estimated whether sociocultural resources (social support, caregiving preparedness, caregiver–patient communication) mediated racial and ethnic differences in caregiver burden.

**Results:**

Compared with non-Hispanic White caregivers (*N* = 1,169), Black (*N* = 220) and Hispanic (*N* = 84) caregivers spent more time caregiving (18 vs. 26 vs. 26 h/week; *P* < 0.001), completed more tasks (6.8 vs. 7.6 vs. 8.7; *P* < 0.05), and reported greater financial burden (*P* = 0.02). Yet, compared to non-Hispanic Whites, Hispanic caregivers reported similar social/emotional and health burdens, while Black caregivers reported lower levels *(P* < 0.01)*.* In adjusted models, disparities in financial burden disappeared, and Hispanic caregivers had less health burden than non-Hispanic White caregivers (*P* = 0.01). Social support and/or caregiving preparedness partially mediated the Black–White gap for all three types of burdens.

**Conclusions:**

Black and Hispanic cancer caregivers perform more caregiving and report greater financial burden than non-Hispanic White caregivers, but experience lower or equivalent social/emotional and health burdens. Racial differences in caregivers’ social support and caregiving preparedness levels partially explain Black–White burden differences. Research and policy should address Black and Hispanic caregivers’ increased financial burden.

**Supplementary Information:**

The online version contains supplementary material available at 10.1007/s00520-022-07367-x.

## Background

Cancer patients rely heavily on friends and family members as caregivers. In addition to transporting patients to treatments and helping to maintain patients’ households, caregivers are essential to patient care, improving patient-provider communication [1], influencing medical decisions [2], managing clinical care needs [3], and emotionally supporting patients [4]. However, this often complex, time-consuming labor can negatively impact caregivers emotionally, socially, physically, and financially [5], with spillover effects for cancer patients’ mental and physical health [6].

Studies of caregiving in non-cancer illnesses (e.g., dementia) find that racial and ethnic minority caregivers perform more hours of caregiving [7], but report less burden than White caregivers [8]. Caregivers from historically marginalized racial and ethnic groups may face higher objective and subjective burdens than non-Hispanic White caregivers (hereafter “White”) due to systemic and interpersonal racism’s effects on physical, mental, and financial health [9, 10]. Indeed, racial and ethnic disparities in cancer care and outcomes are evident across the cancer care trajectory from diagnosis to death [11]. Accordingly, caregivers of racial and ethnic minority backgrounds may experience greater burdens while navigating the medical system and caring for sicker patients.

Historically, marginalized racial and ethnic groups, including those of African and Hispanic descent, hold collectivist cultural ideals (e.g., caring for family and elders) more commonly than White populations, which tend to have more individualistic beliefs and practices [12, 13]. Beliefs and practices associated with collectivist ideals can increase social support and exposure to caregiving, producing better supported and prepared caregivers [7, 14]. These factors may explain why, despite well-documented disparities in healthcare quality and outcomes, most studies find that racial and ethnic minority caregivers report lower or similar levels of emotional burden and stress than White caregivers [7, 15, 16]. Similarly, one study of caregiving’s health impact found an increased risk for White but not Black caregivers’ cardiovascular disease [17], while another found no significant differences in health effects by race and ethnicity [15]. The limited studies of financial burden of caregiving are mixed regarding whether racial and ethnic disparities exist [15, 18, 19].

Less is known, however, about racial and ethnic variation in cancer caregiving duties or how the social/emotional, health, and financial burdens of cancer caregiving may differ by caregivers’ race or ethnicity [20]. Martin et al.’s study examined the same data as this study from the Cancer Care Outcomes Research and Surveillance (CanCORS) study, a large multi-regional study of cancer patients and their caregivers and found that Black caregivers spent more time and performed more caregiving activities than White caregivers [21]. However, Martin et al.’s study did not include Hispanic caregivers, United State’s largest ethnic minority population [22] or explore subjective burden measures. We hypothesized that Hispanic caregivers would also spend more time caregiving and do more tasks than White caregivers. We also extend prior research by investigating subjective burden, hypothesizing that, relative to White caregivers, Black and Hispanic caregivers would report less social/emotional and health burdens. Due to racial and ethnic disparities in wealth, income, and employment [23, 24], however, we hypothesized that Black and Hispanic caregivers would experience more financial burden than White caregivers.

To better inform efforts to address racial and ethnic disparities in caregiving burden, we also built on prior research by investigating targetable resources that might contribute to disparities. Informed by Pearlin’s stress process model [25], we also hypothesized that racial and ethnic differences in caregiving resources would mediate Black–White and Hispanic–White disparities in caregiving burden. Briefly, Pearlin’s model proposes that caregivers’ reactions to stress are shaped by stressors (e.g., patient needs, childcare), sociodemographic factors (e.g., age, gender), and resources (e.g., caregiving preparedness, social support). We also drew upon Dilworth-Anderson’s theoretical extension of this model to consider how racial and ethnic minority groups’ sociocultural contexts (e.g., collectivist orientations, discrimination) may have encouraged resources beneficial for caregiving [26]. For instance, the mobility of families of color has been hindered by racial segregation and economic discrimination, which have also restricted many of these families to the same neighborhoods and multi-generational housing [10]. While these factors compound disadvantages, they can also maintain and facilitate cultural beliefs and practices that protect against burden, like strong knit families and communities [12, 26]. We therefore selected social support, high-quality caregiver–patient communication, and caregiver preparedness as sociocultural resources and examined their potential mediating effects; these resources differ by race and ethnicity, partly due to different social, historical, and cultural factors that racial and ethnic minority and White groups have experienced [21, 27, 28].

## Methods

### Dataset

We used CanCORS Caregiver Study data, a supplemental survey to CanCORS, a longitudinal, multi-regional study of approximately 10,000 patients newly diagnosed with colorectal or lung cancer. Detailed information about CanCORS is published elsewhere [29]. CanCORS randomly selected patients from seven sites nationwide (five population-based cancer registries and two multi-site healthcare systems). Surveys were offered in English and, at the two California sites, also in Spanish, Mandarin, and Cantonese. Participants were representative of newly diagnosed patients with lung and colorectal cancer in US Surveillance, Epidemiology, and End Results Program regions [30]. A subset of patients were asked to elect a caregiver to participate during patients’ baseline (*n* = 827) or follow-up (*n* = 821) interviews. Caregivers were surveyed an average of 7.3 or 16.7 months after the patient’s diagnosis, respectively. All participants provided written informed consent. The study was approved by Dana-Farber’s Office for Human Research Studies (No. 17–294) according to the Belmont Report’s ethical principles.

### Outcome variables

We examined caregiving-related social/emotional, financial, and health burdens (eTable 1). Social/emotional burden was assessed using the Zarit Burden Interview’s modified, fourteen-item version, which measures caregiving-related emotional, social, and relationship stress [31]. For example, caregivers rated their agreement with the statement “My care recipient asks for more help than he/she needs.” Financial burden was measured using three items assessing the impact of caregiving on caregivers’ finances, e.g., “Caring for my care recipient puts a financial strain on me.” Health burden was assessed with a single item from the Zarit Burden Interview: “My health has gotten worse since caring for my care recipient.” Responses were on a five-point Likert scale from “disagree a lot” to “agree a lot.” For social/emotional and financial burden measures, we performed factor analyses with a polychoric correlation matrix to account for ordinal items and used first factors’ loadings (alphas, 0.92 and 0.77, respectively). We re-scaled all burden measures from zero to ten for interpretability. Higher scores indicate greater burden.

### Independent variable

#### Race and ethnicity

Caregivers self-identified as “White,” “Black/African American,” “Asian,” “American Indian/Alaska Native,” “Native Hawaiian,” “Other Pacific Islander,” or “Other.” They were also asked to identify Latino/Hispanic origin (responses: “yes,” “no,” “unsure”). Due to small numbers in several racial groups, we excluded caregivers who identified as other than White, Black/African American or Latino/Hispanic origin (*n* = 95), and those missing data on race/ethnicity (*n* = 27). All caregivers who identified as Latino/Hispanic selected White or Other as their racial category. We excluded two caregivers who identified as Black and Latino/Hispanic origin due to small subgroup size.

### Mediating variables

We hypothesized the following factors could mediate racial and ethnic differences in caregiving burden: social support, caregiving preparedness, and caregiver–patient communication quality (eTable 2).

*Social support* was assessed with the 16-item Medical Outcomes Study (MOS) Social Support Scale, which measures different social support aspects (e.g., emotional support, tangible help, affection) [32]. Responses ranged from “none of the time” to “all of the time” on a 5-point Likert scale. Item responses were averaged for a composite score, ranging from 1 to 5. Higher scores indicate more social support.

*Caregiving preparedness* was assessed with the Family Caregiving Inventory’s four-item subscale, which measures caregivers’ confidence to care for the patient’s emotional and physical needs, find services for the patient, and cope with caregiving stress [33]. Responses ranged from “not at all confident” to “extremely confident” on a 5-point Likert scale. Item responses were averaged for a composite score, with higher scores indicating more preparedness.

*Caregiver–patient communication quality* used caregivers’ response to “How is communication between you and your care recipient these days? In other words, how well can you exchange ideas or talk about things that really concern you right now?” Responses were on a four-point Likert scale from “not at all well” to “very well.” Due to its collinearity with other variables, we collapsed responses into two categories (“not at all well/a little well” vs. “somewhat well/very well”).

### Other covariates

*Caregiving responsibilities* included caregiver-reported hours/week caregiving and number of tasks performed over the past two weeks. Tasks were categorized into activities of daily living (ADLs) (e.g., bathing, dressing), instrumental activities of daily living (IADLs) (e.g., making phone calls, transportation), and clinical care tasks (e.g., monitoring side effects, giving medications). We also assessed primary caregiver status (yes = responsible for at least half of care) and survey timing (i.e., baseline or follow-up).

*Patient clinical factors* included age, stage (I/II or III/IV), and comorbidity severity level using the Adult Comorbidity Evaluation 27 (ACE-27) from medical records and registry data (see Appendices) [34]. Treatment information, collected during patients’ baseline interviews, was not included since treatment status could change prior to caregivers’ surveys.

*Caregiver characteristics* included self-reported age and gender, gender concordance with the patient (e.g., daughter caring for mother), education, and household poverty status (150% of 2005 federal poverty level adjusted for household size) based on caregiver-reported annual household income. We also adjusted for caregivers’ responsibility for children under 18-years-old, employment status, and their relationship to the patient (spouse, child, other). We did not adjust for whether the patient and caregiver lived in the same household since nearly all spouses shared a household with the patient. Additionally, we examined caregivers’ perception of their relationship quality with the patient using a principal component analysis of responses to “Generally how well do you and your care recipient get along together right now?” and “Taking everything into consideration, how close do you feel your relationship is between you and your care recipient right now?” Higher scores indicated higher relationship quality. Lastly, we adjusted for caregiver’s self-rated health (excellent, very good, good, fair, poor).

### Statistical methods

**Fig. 1:**
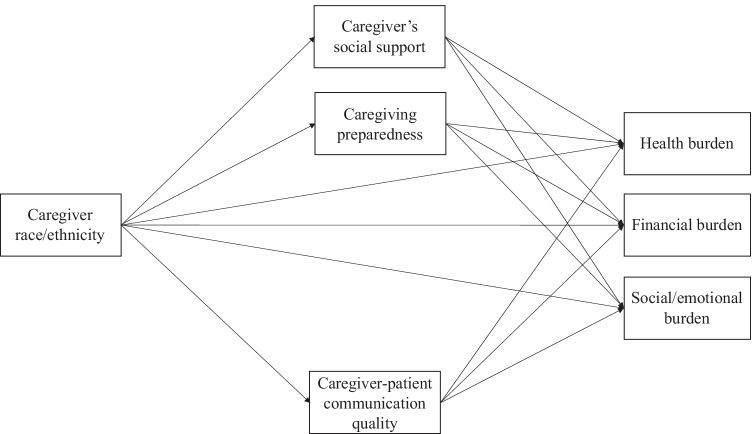
Generalized structural equation model: mediators of caregiver race/ethnicity’s association with burden measures

We estimated *t* tests for all covariates by caregivers’ racial and ethnic group. To investigate factors associated with caregiving burden, we used Stata 16 to fit ordinary least squares regressions for social/emotional and financial burden measures and ordinal logit regression for health burden. To assess whether associations between race/ethnicity and burden measures were mediated by the proposed mediator variables, we estimated a generalized structural equation model using Mplus 8 (Fig. 1) [35]. To estimate standard errors, we used bootstrapping techniques (*b* = 500) with Monte Carlo simulation to adjust for missingness on categorical mediators.

Since missing data ranged from 1 to 12%, we used imputed data for all adjusted analyses except for the generalized structural equation model, which accounts for missingness using maximum likelihood [36]. We used Stata 16’s “mi” multiple imputation procedure to impute twenty datasets. Logistic regression models estimating variable missingness suggested no systematic non-response. We performed sensitivity analyses excluding imputed burden outcomes and found no substantively different results (not shown). Two-sided *P* values < 0.05 were considered statistically significant.

## Results

Among 1473 caregivers, 15% identified as Black (*n* = 220) and 6% as Hispanic (*n* = 84) (eTable 3). Compared to White caregivers, Black caregivers were disproportionately female, younger, employed, responsible for childcare, and economically disadvantaged. Compared to White caregivers, Black caregivers also reported worse health, relatively fewer were the patient’s spouse, and disproportionately more cared for a patient of the same gender (e.g., daughter caring for mother). Black caregivers also reported more social support (*P* = 0.01) and caregiving preparedness (*P* = 0.001) than White caregivers.

Hispanic and White caregivers differed little: Hispanic caregivers were younger and the patients they cared for had more comorbidities (*P* = 0.049) and higher rates of colorectal cancer compared to White caregivers (eTable 3).

### Caregiving responsibilities

**Fig. 2:**
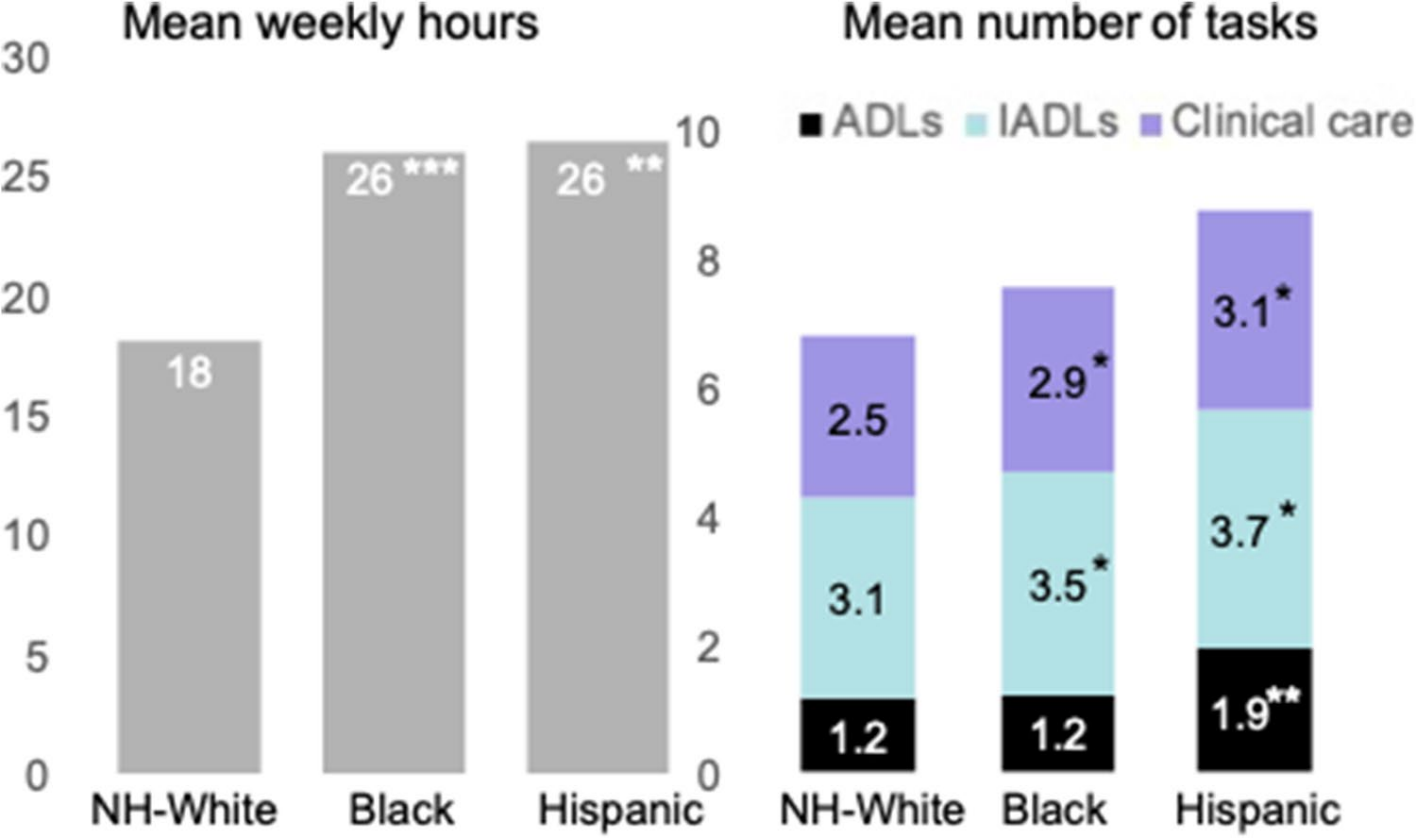
Differences in caregiving responsibilities by caregiver’s race/ethnicity

Black and Hispanic caregivers reported spending more time caregiving and completing more tasks compared to White caregivers. While White caregivers spent 19 hours/week caregiving on average, both Black and Hispanic caregivers reported an average of 26 hours/week (*P* < 0.001 and *P* = 0.006, respectively) (Fig. 2). Compared to White caregivers, Black caregivers completed more IADLs (3.5 vs. 3.1; *P* = 0.03) and clinical care tasks (2.9 vs. 2.5; *P* = 0.03). Similarly, Hispanic caregivers completed more ADLs (1.9 vs. 1.2; *P* = 0.001), IADLs (3.7 vs. 3.1; *P* = 0.03), and clinical care tasks (3.1 vs. 2.5; *P* = 0.02) than White caregivers.

### Caregiver burden

Although Black caregivers devoted more time than White caregivers to caregiving and completed more tasks, they reported lower caregiving-related social/emotional (*P* = 0.002) (Table 1) and health burdens (*P* = 0.002), which were approximately a quarter of a standard deviation lower. There were no statistical differences in social/emotional and health burdens between Hispanic and White caregivers. Compared to White caregivers, Black (*P* = 0.02) and Hispanic (*P* = 0.01) caregivers reported greater financial burdens of a magnitude of 18% and 27% of a standard deviation, respectively.

**Table 1:** Unadjusted and adjusted associations with caregiver social/emotional, financial, and health burdens (*N* = 1473)

	Social/emotional burden			Financial burden			Health burden		
*Unadjusted*	*Mean*	*95% CI*	*P*	*Mean*	*95% CI*	*P*	*Mean*	*95% CI*	*P*
Non-Hispanic White (ref)	4.6	4.5, 4.7		4.2	4.1, 4.3		2.5	2.3, 2.7	
Black	4.2	4.0, 4.4	0.002	4.5	4.3, 4.7	0.02	2.5	1.4, 2.2	0.002
Hispanic	4.8	4.4, 5.1	0.39	4.6	4.3, 5.0	0.02	2.2	1.6, 2.8	0.36
*Adjusted*^a^	*Beta*	*95% CI*	*P*	*Beta*	*95% CI*	*P*	*Beta*	*95% CI*	*P*
Caregiver race/ethnicity (ref: non-Hispanic White)									
Black	− 0.45	− 0.65, − 0.25	0.00	0.05	− 0.17, 0.27	0.66	− 0.80	− 1.15, − 0.45	0.00
Hispanic	− 0.15	− 0.45, 0.15	0.32	0.27	− 0.07, 0.60	0.12	− 0.62	− 1.10, − 0.14	0.01
Mediators									
Caregiver–patient communication quality (ref = not well at all/a little well)									
Somewhat well/very well	− 0.29	− 0.53, − 0.05	0.02	0.00	− 0.27, 0.26	0.98	0.02	− 0.34, 0.37	0.93
Social support	− 0.32	− 0.40, − 0.24	0.00	− 0.25	− 0.34, − 0.16	0.00	− 0.30	− 0.43, − 0.17	0.00
Caregiving preparedness	− 0.36	− 0.46, − 0.26	0.00	− 0.14	− 0.25, − 0.03	0.01	− 0.31	− 0.46, − 0.15	0.00

Estimated with imputed data.

^a^Adjusted for of sociodemographic, clinical, and caregiving factors. Social/emotional and financial burden estimated with OLS regression. Health burden estimated with binary logit regression.

#### Social/emotional burden

In models adjusted for patient clinical factors, caregiving responsibilities, caregiver sociodemographic, and psychosocial mediators, identifying as Black was associated with lower social/emotional burden compared to identifying as White (β =  − 0.44; *P* < 0.001) (Table 1). Among hypothesized mediators, caregiver–patient communication quality (β =  − 0.29; *P* = 0.02), caregivers’ social support (β =  − 0.30; *P* < 0.001), and caregiving preparedness (β =  − 0.34; *P* < 0.001) were associated with lower burden. See Appendices for full model results (eTable 4).

#### Financial burden

After adjustment for the aforementioned factors, Black–White and Hispanic–White gaps in financial burden were no longer statistically significant. Among the proposed mediators, greater social support (β =  − 0.26; *P* < 0.001) and caregiving preparedness (β =  − 0.13; *P* = 0.03) were statistically associated with lower financial burden.

#### Health burden

After adjustment, identifying as Black (β =  − 0.77; *P* < 0.001) or Hispanic (β =  − 0.63; *P* = 0.01) was associated with statistically significantly lower health burden than White caregivers. Among the mediators, social support (β =  − 0.28; *P* < 0.001) and caregiving preparedness (β =  − 0.29; *P* < 0.001) were statistically associated with less burden.

#### Mediation

Mediation model (Fig. 1) results indicated that two hypothesized mediators, social support and caregiving preparedness, partially explained Black–White gaps in all burden types, but no mediators explained the Hispanic–White gaps. Relative to White caregivers, Black caregivers’ social support reduced their social/emotional burden levels by 0.08 (*P* = 0.01) (Table 2), and their caregiving preparedness reduced the burden by 0.12 points (*P* < 0.001). Together, these indirect effects accounted for 21% of the Black–White gap in social/emotional burden. Black race’s direct effect on social/emotional burden explained most of the gap (76%; *P* < 0.001).

**Table 2:** Structural equation model estimating effects of potential mediators of caregiver race/ethnicity’s association with burden measures (*N* = 1126)

	Social/emotional burden				Financial burden				Health burden			
	% total effect	Effect	95% CI	*P*	% total effect	Effect	95% CI	*P*	% total effect	Effect	95% CI	*P*
Black caregivers (vs. White)												
Social support	9%	− 0.08	− 0.15, − 0.04	0.01	115%	− 0.11	− 0.19, − 0.05	0.02	5%	− 0.06	− 0.13, − 0.02	0.05
Caregiving preparedness	12%	− 0.12	− 0.19, − 0.07	0.001	88%	− 0.08	− 0.15, − 0.03	0.03	8%	− 0.09	− 0.16, − 0.04	0.02
Communication quality	3%	− 0.03	− 0.05, 0.00	0.10	− 14%	0.01	− 0.02, 0.04	0.57	1%	− 0.01	− 0.06, − 0.02	0.49
Direct effect	76%	− 0.73	− 0.92, − 0.47	0.00	− 89%	0.08	− 0.34, 0.51	0.75	85%	− 0.91	− 1.28, − 0.51	0.00
Hispanic caregivers (vs. White)												
Social support	3%	− 0.01	− 0.09, 0.06	0.83	− 29%	− 0.01	− 0.11, 0.08	0.83	1%	− 0.01	− 0.07, 0.05	0.84
Caregiving preparedness	16%	− 0.05	− 0.14, 0.03	0.36	− 94%	− 0.03	− 0.11, 0.02	0.40	5%	− 0.04	− 0.12, 0.02	0.41
Communication quality	2%	− 0.01	− 0.04, 0.02	0.56	11%	0.00	− 0.01, 0.04	0.77	1%	− 0.01	− 0.04, 0.02	0.71
Direct effect	78%	− 0.23	− 0.67, 0.19	0.38	211%	0.07	− 0.54, 0.68	0.84	94%	− 0.73	− 1.35, − 0.18	0.03

*Non-imputed data; models adjusted for caregiver and patient demographics, patient clinical factors, and caregiving characteristics; percent of total effect may not sum to 100 due to inconsistent mediation.

While Black caregivers reported greater financial burdens than White caregivers, mediation models indicated that social support reduced Black caregivers’ financial burden relative to White caregivers’ by 0.11 points (*P* = 0.02) and caregiving preparedness reduced Black caregivers’ burden by 0.08 points (*P* = 0.03). Since social support’s and caregiving preparedness’s effects on financial burden were in the opposite direction of Black race’s effect (i.e., Black race was associated with higher financial burden, whereas social support and preparedness were associated with less financial burden), we could not approximate the extent that social support and preparedness accounted for financial burden’s Black–White disparity.

Regarding health burden, Black caregivers experienced lower levels than White caregivers. Greater caregiving preparedness among Black individuals (*P* = 0.02) accounted for 8% of the Black–White gap and social support accounted for 6% (*P* = 0.05), while Black race’s direct effect on health burden accounted for 85% of the gap (*P* < 0.001). The direct effect of being Hispanic accounted for 94% of the reduction (*P* = 0.03).

## Discussion

Our findings indicate that Black and Hispanic cancer caregivers devote more time to caregiving, complete more caregiving tasks, and report greater financial burdens than White cancer caregivers. However, Black caregivers report lower social/emotional and health burdens than White caregivers, while Hispanic caregivers report burden levels similar to White caregivers. We found some evidence that racial and ethnic differences in resources—social support and caregiving preparedness—partially mediated racial disparities in caregiving burden. That is, Black caregivers’ relatively higher levels of caregiving preparedness and social support mitigated their social/emotional, financial, and health burdens from caregiving compared to White caregivers. These findings also suggest that the Black–White disparity in financial caregiving would be even greater absent Black caregivers’ increased levels of social support and caregiving preparedness.

Our findings build on Martin et al.’s prior study using CanCORS data [21] by including Hispanic cancer caregivers who are relatively understudied in the cancer caregiver literature. Our study is one of the first to examine racial and ethnic differences in cancer caregivers’ subjective burdens. Our findings that Black caregivers report less social/emotional burdens than White caregivers parallel those from studies of non-cancer specific caregivers [7, 37]. We extend prior research, however, by considering whether racial and ethnic differences in social- and culture-influenced resources—social support, caregiving preparedness, and communication quality—mediate disparities in burden.

### Clinical implications

Our study indicates that Black caregivers have greater sociocultural resources than White caregivers, which helps reduce Black caregivers’ burden. These findings have several potential implications for practice. First, they suggest that caregivers may benefit from psychosocial interventions designed to improve caregiving preparedness and social support. Our findings also suggest that, although racial and ethnic minority caregivers may report less caregiving burden than White caregivers, their burden levels remain substantial. For Black caregivers, structural forces that have simultaneously disadvantaged the Black population may have paradoxically increased social support and caregiving preparedness. For example, economic and housing discrimination have created racially segregated neighborhoods where many Black families have remained for decades in multi-generational housing [10]. The US healthcare system has long discriminated against Black patients and families, fostering medical mistrust [38]. This may have encouraged Black families to rely on personal networks and filial obligation, ultimately increasing their social support and experience with caregiving than White caregivers [7, 14, 19].

Our findings that Black and Hispanic caregivers experienced more financial burden than White caregivers add to extensive research demonstrating that Black and Hispanic individuals are, on average, economically disadvantaged on every metric compared to White individuals [23]. The average White family has approximately ten times more wealth than the average Black and Hispanic families [39]. Consequently, our research supports prioritizing Black and Hispanic families for supportive services like financial distress screening, connecting families to financial resources, and familiarizing them with insurance plan limitations and the need to plan childcare or caregiving coverage [40]. At a policy level, our study supports expanding access to paid family leave, a goal of President Biden’s proposed American Families Plan [41]. Future research could explore possible benefits of direct financial support for caregiving, similar to the Department of Veteran Affairs’ Program of Comprehensive Assistance for Family Caregivers, which includes compensating family caregivers for care [42].

### Study limitations

While these data are older (2003–2007), CanCORS is one of the few multi-regional studies of cancer caregivers that is sufficiently large to study racial and ethnic differences across a variety of burden measures and mediators. New data collection efforts are needed, however, since, due to sample numbers, we could not include other historically marginalized racial and ethnic groups. Efforts are sorely needed to collect data from these groups like Indigenous Americans and Asian Americans and their subgroups to examine the extent and sources of disparities. Since our analyses only partially explained disparities, other variables also may mediate the association between race and ethnicity and caregiving burden. We only explored caregiving-related factors that we theorized were potential intervention targets, such as identifying and using resources like friends, family members, and community services and developing caregiving skills. More structural (and thus more immutable) forces likely contribute to financial burden disparities, like workplace flexibility, which benefits White caregivers more often than other racial groups [43]. Similarly, as outlined by our conceptual model, sociocultural resources vary by racial and ethnic identity, differentially shaping attitudes towards and experiences of caregiving in many ways that could explain Black and Hispanic caregivers’ lower caregiving burdens. Relatedly, dementia caregiving researchers have questioned whether burden instruments are culturally relevant for Black caregivers and thus can accurately measure their burden level [44]. While some have validated burden measures for different ethnic groups, mixed-method work is needed. We also were unable to control for English language skills, which may particularly influence the ability of some caregivers of color to navigate the healthcare system. Similarly, we could not adjust for immigrant status, which could affect the Hispanic–White gaps in social/emotional and health burdens. Studies of immigrant assimilation and the Hispanic health paradox find that Hispanic cultural values, which emphasize caring for family elders, become “Americanized” and health outcomes worsen as more family generations are US-born [12], which could impact caregiving expectations and abilities.

## Conclusion

Our study indicates that, while Black and Hispanic family caregivers devote more time to caregiving for a patient with cancer and report greater financial burden than non-Hispanic White caregivers, they report less or equivalent social, emotional, and health burdens. Our findings indicate that Black caregivers’ increased social support and caregiving preparedness mediated the Black–White gaps in caregiving burdens. This suggests that sociocultural resources that are beneficial to caregiving may benefit other caregivers since they can be strengthened via psychosocial interventions. Future research, healthcare resources, and policy should be devoted to ameliorating Black and Hispanic caregivers’ increased financial burden.

## Supplementary Information

Below is the link to the electronic supplementary material.

Supplementary file1 (DOCX 72 KB)
