# Supplementary material for: Racial and ethnic disparities in cancer caregiver burden and potential sociocultural mediators
Source: Support Care Cancer. 2022 Oct 3;30(11):9625–33. doi: 10.1007/s00520-022-07367-x (PMC9633462; doi:10.1007/s00520-022-07367-x)
Supplement: Supplementary file 1 — Supplementary file1 (DOCX 72 KB) [file 520_2022_7367_MOESM1_ESM.docx]

# **Appendix**

The ACE-27 measure categorizes specific diseases and conditions based on the following levels of severity: none, grade 1 (mild), grade 2 (moderate), or grade 3 (severe). Severity is based on a morbidity’s prognostic impact or individual organ decompensation. An overall comorbidity score (none, mild, moderate, or severe) is determined based on an individual’s most severely ranked morbidity. When an individual has multiple morbidities ranked as ‘moderate’ in different organ systems or disease groupings, the overall score is assessed as ‘severe.’

| **eTable 1.** Factor loadings for survey items in the factor analysis of social/emotional and financial burden measures | | |
| --- | --- | --- |
| Items | Emotional/Social | Financial |
| I feel stressed between caring for my Care Recipient and trying to meet other responsibilities for my family or work | 0.79 |  |
| I have lost control of my life since my Care Recipient’s illness | 0.85 |  |
| I feel very burdened by having to care for my Care Recipient | 0.82 |  |
| I feel that my Care Recipient affects my relationships with family members or friends in a negative way | 0.77 |  |
| My health has gotten worse since caring for my Care Recipient | 0.72 |  |
| Because of the time I spend with my Care Recipient I don’t have enough time for myself | 0.83 |  |
| I worry that I won’t be able to do enough | 0.50 |  |
| I feel uncertain about what to do about my Care Recipient | 0.67 |  |
| I worry that I’ll have to give up more and more things in the future | 0.77 |  |
| My social life has suffered because I am caring for my Care Recipient | 0.82 |  |
| I don’t have as much privacy as I would like because of my Care Recipient | 0.82 |  |
| My Care Recipient seems to expect me to take care of him/her as if I were the only one he or she could depend on | 0.74 |  |
| I wish I could leave the care of my Care Recipient to someone else | 0.74 |  |
| My Care Recipient asks for more help than he/she needs | 0.63 |  |
| My financial resources are adequate to pay for things that are required for caregiving |  | 0.62 |
| It is difficult to pay for the things my Care Recipient needs |  | 0.87 |
| Caring for my Care Recipient puts a financial strain on me |  | 0.83 |
| *Proportion of variance explained* | 0.91 | 1.12 |
| ^1^Responses ranged from 'disagree a lot' to 'agree a lot' on 5-point Likert scale | | |

| **eTable 2.** Factor loadings for survey items in the factor analysis of social support, caregiving preparedness, and positive aspects of caregiving measures | |
| --- | --- |
| Measures | Factor loadings |
| Social support [How often do you have…] |  |
| Someone to help you if you were confined to bed | 0.22 |
| Someone to take you to the doctor if you needed it | 0.21 |
| Someone to prepare your meals if you were unable to do it yourself | 0.24 |
| Someone to help with daily chores if you were sick | 0.24 |
| Someone you can count on to listen to you when you need to talk | 0.26 |
| Someone to give you good advice in a crisis | 0.26 |
| Someone to give you information to help you understand a situation | 0.26 |
| Someone to confide in or talk to about yourself or your problems | 0.27 |
| Someone to share your most private worries and fears with | 0.27 |
| Someone whose advice you really want | 0.26 |
| Someone to do something enjoyable with | 0.26 |
| Someone who understands your problems | 0.27 |
| Someone who shows you love and affection | 0.24 |
| Someone to love you and make you feel wanted | 0.23 |
| Someone to have a good time with | 0.25 |
| Someone to get together with for relaxation | 0.26 |
| Caregiving preparedness [How confident are you that you can…] |  |
| Take care of your Care Recipient’s physical needs? | 0.50 |
| Take care of your Care Recipient’s emotional needs? | 0.51 |
| Find out about services for your Care Recipient? | 0.47 |
| Cope with the stress of caregiving? | 0.51 |

|  | **eTable 3**. Caregiver and patient characteristics by caregiver race/ethnicity | | | | | | | | | | | | | | | |  |
| --- | --- | --- | --- | --- | --- | --- | --- | --- | --- | --- | --- | --- | --- | --- | --- | --- | --- |
|  | |  | All (N=1473) | |  | Non-Hispanic White (N=1169) | |  | Black  (N=220) | | |  | Hispanic   (N=84) | | | | |
|  | | Missing | N | % |  | N | % |  | N | % | P |  | | N | % | P | |
| **Caregiver characteristics** | |  |  |  |  |  |  |  |  |  |  |  | |  |  |  | |
| Gender | |  |  |  |  |  |  |  |  |  |  |  | |  |  |  | |
| Female | | 8 | 1111 | 75.4 |  | 863 | 73.8 |  | 184 | 83.6 | 0.002 |  | | 64 | 76.2 | 0.62 | |
| Male | |  | 362 | 24.6 |  | 306 | 26.2 |  | 36 | 16.4 |  |  | | 20 | 23.8 |  | |
| Age (mean, SD) | | 0 | 57.0 | 13.2 |  | 58.1 | 12.9 |  | 53.0 | 12.7 | 0.00 |  | | 52.4 | 15.5 | 0.00 | |
| Relation to patient | |  |  |  |  |  |  |  |  |  |  |  | |  |  |  | |
| Spouse/partner | | 8 | 935 | 63.5 |  | 771 | 65.9 |  | 115 | 52.4 | 0.00 |  | | 49 | 58.1 | 0.33 | |
| Child | |  | 208 | 14.1 |  | 159 | 13.6 |  | 31 | 14.3 |  |  | | 17 | 20.5 |  | |
| Other | |  | 330 | 22.4 |  | 239 | 20.5 |  | 73 | 33.3 |  |  | | 18 | 21.4 |  | |
| Educational attainment | |  |  |  |  |  |  |  |  |  |  |  | |  |  |  | |
| Some college or higher | | 9 | 947 | 64.3 |  | 748 | 64.0 |  | 139 | 63.3 | 0.82 |  | | 60 | 71.0 | 0.20 | |
| High school degree or less | |  | 526 | 35.7 |  | 421 | 36.0 |  | 81 | 36.7 |  |  | | 24 | 29.0 |  | |
| Household federal poverty threshold | |  |  |  |  |  |  |  |  |  |  |  | |  |  |  | |
| Below 150% | | 1 | 270 | 18.3 |  | 191 | 16.3 |  | 61 | 27.7 | 0.00 |  | | 18 | 21.4 | 0.24 | |
| At least 150% | |  | 1203 | 81.7 |  | 978 | 83.7 |  | 159 | 72.3 |  |  | | 66 | 78.6 |  | |
| Employment status | |  |  |  |  |  |  |  |  |  |  |  | |  |  |  | |
| Part-time / full-time | | 79 | 751 | 51.0 |  | 581 | 49.7 |  | 128 | 58.0 | 0.04 |  | | 42 | 50.0 | 0.97 | |
| No paid work | |  | 722 | 49.0 |  | 588 | 50.3 |  | 92 | 42.0 |  |  | | 42 | 50.0 |  | |
| Responsible for children under 18-years-old | |  |  |  |  |  |  |  |  |  |  |  | |  |  |  | |
| Yes | | 0 | 238 | 16.1 |  | 164 | 14.0 |  | 60 | 27.3 | 0.00 |  | | 14 | 16.7 | 0.58 | |
| No | |  | 1235 | 83.9 |  | 1005 | 86.0 |  | 160 | 72.7 |  |  | | 70 | 83.3 |  | |
|  | |  |  |  |  |  |  |  |  |  |  |  | |  |  |  | |
|  | |  |  |  |  |  |  |  |  |  |  |  | |  |  |  | |
|  | |  |  |  |  |  |  |  |  |  |  |  | |  |  |  | |

|  |  | All (N=1473) | |  | Non-Hispanic White (N=1169) | |  | Black  (N=220) | | |  | Hispanic   (N=84) | | |
| --- | --- | --- | --- | --- | --- | --- | --- | --- | --- | --- | --- | --- | --- | --- |
|  | Missing | N | % |  | N | % |  | N | % | P |  | N | % | P |
| Self-rated physical health |  |  |  |  |  |  |  |  |  |  |  |  |  |  |
| Excellent | 11 | 150 | 10.2 |  | 129 | 11.1 |  | 13 | 5.9 | 0.02 |  | 7 | 8.4 | 0.51 |
| Very good |  | 472 | 32.0 |  | 380 | 32.5 |  | 64 | 29.2 |  |  | 27 | 32.5 |  |
| Good |  | 549 | 37.3 |  | 425 | 36.4 |  | 92 | 42.0 |  |  | 31 | 37.4 |  |
| Fair |  | 266 | 18.1 |  | 206 | 17.6 |  | 44 | 20.1 |  |  | 16 | 19.3 |  |
| Poor |  | 37 | 2.5 |  | 29 | 2.5 |  | 6 | 2.7 |  |  | 2 | 2.4 |  |
| **Caregiving characteristics** |  |  |  |  |  |  |  |  |  |  |  |  |  |  |
| Primary caregiver |  |  |  |  |  |  |  |  |  |  |  |  |  |  |
| Yes | 77 | 1258 | 85.4 |  | 999 | 85.5 |  | 184 | 83.6 | 0.46 |  | 74 | 88.6 | 0.42 |
| No |  | 215 | 14.6 |  | 170 | 14.5 |  | 36 | 16.4 |  |  | 10 | 11.4 |  |
| Interview wave |  |  |  |  |  |  |  |  |  |  |  |  |  |  |
| Follow-up | 0 | 723 | 49.1 |  | 566 | 48.4 |  | 104 | 47.2 | 0.74 |  | 53 | 63.1 | 0.01 |
| Baseline |  | 750 | 50.9 |  | 603 | 51.6 |  | 116 | 52.8 |  |  | 31 | 36.9 |  |
| **Caregiver-patient relationship characteristics** |  |  |  |  |  |  |  |  |  |  |  |  |  |  |
| Gender concordance |  |  |  |  |  |  |  |  |  |  |  |  |  |  |
| Yes | 9 | 337 | 22.9 |  | 244 | 20.8 |  | 70 | 31.8 | 0.00 |  | 24 | 28.6 | 0.10 |
| No |  | 1136 | 77.1 |  | 925 | 79.2 |  | 150 | 68.2 |  |  | 60 | 71.4 |  |
| Caregiver-patient relationship quality (mean, SD) | 54 | 3.5 | 0.5 |  | 3.5 | 0.5 |  | 3.5 | 0.5 | 0.53 |  | 3.5 | 0.5 | 0.29 |
| **Patient characteristics** |  |  |  |  |  |  |  |  |  |  |  |  |  |  |
| Gender |  |  |  |  |  |  |  |  |  |  |  |  |  |  |
| Female | 21 | 546 | 37.1 |  | 427 | 36.5 |  | 84 | 38.2 | 0.67 |  | 36 | 42.9 | 0.26 |
| Male |  | 927 | 62.9 |  | 742 | 63.5 |  | 136 | 61.8 |  |  | 48 | 57.1 |  |
| Patient age (mean, SD) | 1 | 63.4 | 17.1 |  | 64.5 | 16.7 |  | 58.5 | 17.2 | 0.00 |  | 61.2 | 19.6 | 0.08 |
| Cancer type |  |  |  |  |  |  |  |  |  |  |  |  |  |  |
| Colon | 1 | 769 | 52.2 |  | 574 | 49.1 |  | 139 | 63.2 | 0.00 |  | 56 | 66.7 | 0.002 |
| Lung |  | 704 | 47.8 |  | 595 | 50.9 |  | 81 | 36.8 |  |  | 28 | 33.3 |  |

|  |  | All (N=1473) | |  | | Non-Hispanic White (N=1169) | | | |  | | Black  (N=220) | | | | | |  | Hispanic   (N=84) | | | | | |
| --- | --- | --- | --- | --- | --- | --- | --- | --- | --- | --- | --- | --- | --- | --- | --- | --- | --- | --- | --- | --- | --- | --- | --- | --- |
|  | Missing | N | % |  | | N | | | % |  | | N | | % | | P | |  | N | | % | | P | |
| Stage at diagnosis |  |  |  |  | |  | | |  |  | |  | |  | |  | |  |  | |  | |  | |
| Stage III/IV | 59 | 745 | 50.6 |  | | 586 | | | 50.2 |  | | 116 | | 52.8 | | 0.53 | |  | 42 | | 50.4 | | 1.00 | |
| Stage I/II |  | 728 | 49.4 |  | | 583 | | | 49.8 |  | | 104 | | 47.3 | |  | |  | 42 | | 49.6 | |  | |
| Comorbidity level |  |  |  |  | |  | | |  |  | |  | |  | |  | |  |  | |  | |  | |
| None | 176 | 369 | 25.1 |  | | 274 | | | 23.4 |  | | 68 | | 30.8 | | 0.002 | |  | 29 | | 35.0 | | 0.049 | |
| Grade 1, mild |  | 553 | 37.6 |  | | 432 | | | 37.0 |  | | 89 | | 40.5 | |  | |  | 32 | | 38.3 | |  | |
| Grade 2, moderate |  | 303 | 20.6 |  | | 247 | | | 21.1 |  | | 43 | | 19.5 | |  | |  | 13 | | 15.0 | |  | |
| Grade 3, severe |  | 248 | 16.8 |  | | 216 | | | 18.5 |  | | 20 | | 9.2 | |  | |  | 10 | | 11.7 | |  | |
| **Caregiving mediators** |  |  |  |  | |  | | |  |  | |  | |  | |  | |  |  | |  | |  | |
| Caregiver-patient communication quality |  |  |  |  | |  | | |  |  | |  | |  | |  | |  |  | |  | |  | |
| Not well at all/A little well | 15 | 218 | 14.8 |  | | 177 | | | 15.1 |  | | 28 | | 12.8 | | 0.39 | |  | 13 | | 15.6 | | 0.90 | |
| Very well/Somewhat well |  | 1255 | 85.2 |  | | 992 | | | 84.9 |  | | 192 | | 87.2 | |  | |  | 71 | | 84.4 | |  | |
| Support (mean, SD) | 31 | 3.8 | 0.9 |  | | 3.8 | | | 0.9 |  | | 3.9 | | 0.9 | | 0.01 | |  | 3.8 | | 0.9 | | 0.89 | |
| Caregiving preparedness (mean, SD) | 23 | 3.6 | 0.8 |  | | 3.6 | | | 0.8 |  | | 3.8 | | 0.8 | | 0.001 | |  | 3.6 | | 0.7 | | 0.83 | |
| *Non-adjusted imputed data | | | | |  | |  |  | | |  | |  | |  | |  | |  |  | |  | |  |

| **eTable 4.** Adjusted associations of sociodemographic, clinicial, and caregiving factors with caregiver social/emotional, financial, and health burdens (N=1473) | | | | | | | | | | | | | | | | | | | | | | | | | | | | | | | | | | | | | | |
| --- | --- | --- | --- | --- | --- | --- | --- | --- | --- | --- | --- | --- | --- | --- | --- | --- | --- | --- | --- | --- | --- | --- | --- | --- | --- | --- | --- | --- | --- | --- | --- | --- | --- | --- | --- | --- | --- | --- |
|  | Social/emotional burden^1^ | | | | | |  | | Financial burden^1^ | | | | | | | | | | | |  | | | | Health burden^2^ | | | | | | | | | | | |  |  |
|  | Beta | 95% CI | | P | | |  | | Beta | | 95% CI | | | | P | | | |  | | | | Beta | | | | 95% CI | | | | P | | | |  |  |  |  |
| **Caregiver race/ethnicity** (ref: Non-Hispanic White) | | | | | | |  |  | | | |  | | | |  | | | |  | | | |  | | | |  | | | |  | | | |  |  |  |
| Black | -0.45 | -0.65, -0.25 | | 0.00 | | |  | | 0.05 | | | | -0.17, 0.27 | | | | 0.66 | | | |  | | | | -0.80 | | | | -1.15, -0.45 | | | | 0.00 | | | |  |  |
|  |  |  | |  | | |  | |  | | | |  | | | |  | | | |  | | | |  | | | |  | | | |  | | | |  |  |
| Hispanic | -0.15 | -0.45, 0.15 | | 0.32 | | |  | | 0.27 | | | | -0.07, 0.60 | | | | 0.12 | | | |  | | | | -0.62 | | | | -1.10, -0.14 | | | | 0.01 | | | |  |  |
| ***Patient clinical factors*** |  |  | |  | | |  | |  | | | |  | | | |  | | | |  | | | |  | | | |  | | | |  | | | |  |  |
| Age | 0.01 | 0.00, 0.01 | | 0.003 | | |  | | -0.01 | | | | -0.01, 0.00 | | | | 0.02 | | | |  | | | | 0.00 | | | | -0.01, 0.01 | | | | 0.72 | | | |  |  |
| Cancer type (ref: Lung) |  |  | |  | | |  | |  | | | |  | | | |  | | | |  | | | |  | | | |  | | | |  | | | |  |  |
| Colon | 0.04 | -0.10, 0.18 | | 0.57 | | |  | | -0.03 | | | | -0.18, 0.12 | | | | 0.70 | | | |  | | | | -0.10 | | | | -0.33, 0.12 | | | | 0.38 | | | |  |  |
| Stage at diagnosis (ref: Stage I/II) | | |  | |  | |  |  | | | |  | | | |  | | | |  | | | |  | | | |  | | | |  | | | |  |  |  |
| Stage III/IV | 0.11 | -0.25, 0.03 | | 0.13 | | |  | | 0.01 | | | | -0.14, 0.17 | | | | 0.87 | | | |  | | | | 0.14 | | | | -0.09, 0.37 | | | | 0.24 | | | |  |  |
| Comorbidity level (ref: None) |  |  | |  | | |  | |  | | | |  | | | |  | | | |  | | | |  | | | |  | | | |  | | | |  |  |
| Grade 1, mild | -0.11 | -0.30, 0.07 | | 0.23 | | |  | | 0.06 | | | | -0.14, 0.27 | | | | 0.53 | | | |  | | | | -0.12 | | | | -0.43, 0.19 | | | | 0.45 | | | |  |  |
|  |  |  | |  | | |  | |  | | | |  | | | |  | | | |  | | | |  | | | |  | | | |  | | | |  |  |
| Grade 2, moderate | -0.07 | -0.29, 0.15 | | 0.53 | | |  | | 0.10 | | | | -0.13, 0.33 | | | | 0.40 | | | |  | | | | -0.34 | | | | -0.70, 0.02 | | | | 0.07 | | | |  |  |
|  |  |  | |  | | |  | |  | | | |  | | | |  | | | |  | | | |  | | | |  | | | |  | | | |  |  |
| Grade 3, severe | 0.06 | -0.18, 0.31 | | 0.62 | | |  | | 0.11 | | | | -0.15, 0.38 | | | | 0.40 | | | |  | | | | -0.31 | | | | -0.70, 0.08 | | | | 0.12 | | | |  |  |
| ***Caregiving characteristics*** | | |  | |  | |  |  | | | |  | | | |  | | | |  | | | |  | | | |  | | | |  | | | |  |  |  |
| Hours/week caregiving | 0.00 | 0.00, 0.01 | | 0.03 | | |  | | 0.00 | | | | 0.00, 0.01 | | | | 0.16 | | | |  | | | | 0.00 | | | | -0.01, 0.00 | | | | 0.62 | | | |  |  |
|  |  |  | |  | | |  | |  | | | |  | | | |  | | | |  | | | |  | | | |  | | | |  | | | |  |  |
| Number of ADLs performed | 0.06 | 0.02, 0.11 | | 0.003 | | |  | | -0.01 | | | | -0.05, 0.04 | | | | 0.82 | | | |  | | | | 0.03 | | | | -0.04, 0.09 | | | | 0.38 | | | |  |  |
|  |  |  | |  | | |  | |  | | | |  | | | |  | | | |  | | | |  | | | |  | | | |  | | | |  |  |
| Number of IADLs performed | 0.15 | 0.11, 0.19 | | 0.00 | | |  | | 0.09 | | | | 0.04, 0.13 | | | | 0.00 | | | |  | | | | 0.13 | | | | 0.07, 0.19 | | | | 0.00 | | | |  |  |
|  |  |  | |  | | |  | |  | | | |  | | | |  | | | |  | | | |  | | | |  | | | |  | | | |  |  |
| Number of clinical care tasks performed | 0.09 | 0.04, 0.13 | | 0.00 | | |  | | 0.03 | | | | -0.02, 0.09 | | | | 0.18 | | | |  | | | | 0.14 | | | | 0.07, 0.21 | | | | 0.00 | | | |  |  |
|  | Social/emotional burden | | | | | |  | | Financial burden | | | | | | | | | | | |  | | | | Health burden | | | | | | | | | | | |  |  |
|  | Beta | 95% CI | | P | | |  | | Beta | | | | 95% CI | | | | P | | | |  | | | | Beta | | | | 95% CI | | | | P | | | |  |  |
| Primary caregiver (ref: No) |  |  | |  | | |  | |  | | | |  | | | |  | | | |  | | | |  | | | |  | | | |  | | | |  |  |
| Yes | 0.15 | -0.06, 0.37 | | 0.15 | | |  | | -0.05 | | | | -0.20, 0.11 | | | | 0.58 | | | |  | | | | -0.02 | | | | -0.39, 0.33 | | | | 0.90 | | | |  |  |
| Survey timing (ref: Baseline) | | |  | |  | |  |  | | | |  | | | |  | | | |  | | | |  | | | |  | | | |  | | | |  |  |  |
| Follow-up | 0.10 | -0.04, 0.24 | | 0.15 | | |  | | -0.04 | | | | -0.20, 0.11 | | | | 0.58 | | | |  | | | | 0.28 | | | | 0.05, 0.50 | | | | 0.02 | | | |  |  |
| ***Caregiver characteristics*** | | |  | |  | |  |  | | | |  | | | |  | | | |  | | | |  | | | |  | | | |  | | | |  |  |  |
| Gender (ref=male) |  |  | |  | | |  | |  | | | |  | | | |  | | | |  | | | |  | | | |  | | | |  | | | |  |  |
| Female | 0.31 | 0.14, 0.48 | | 0.00 | | |  | | 0.26 | | | | 0.08, 0.44 | | | | 0.01 | | | |  | | | | 0.56 | | | | 0.27, 0.85 | | | | 0.00 | | | |  |  |
| Relation to patient (ref: Spouse) | | |  | |  | |  |  | | | |  | | | |  | | | |  | | | |  | | | |  | | | |  | | | |  |  |  |
| Child | 0.13 | -0.13, 0.39 | | 0.34 | | |  | | -0.01 | | | | -0.30, 0.27 | | | | 0.92 | | | |  | | | | -0.45 | | | | -0.86, -0.03 | | | | 0.04 | | | |  |  |
|  |  |  | |  | | |  | |  | | | |  | | | |  | | | |  | | | |  | | | |  | | | |  | | | |  |  |
| Other | -0.03 | -0.25, 0.20 | | 0.82 | | |  | | -0.05 | | | | -0.29, 0.20 | | | | 0.70 | | | |  | | | | -0.58 | | | | -0.82, -0.08 | | | | 0.02 | | | |  |  |
| Age (ref: 65-years-old or less) | | |  | |  | |  |  | | | |  | | | |  | | | |  | | | |  | | | |  | | | |  | | | |  |  |  |
| Over 65-years-old | -0.23 | -0.42, -0.05 | | 0.01 | | |  | | -0.27 | | | | -0.47, -0.07 | | | | 0.01 | | | |  | | | | -0.28 | | | | -0.57, 0.02 | | | | 0.07 | | | |  |  |
|  |  |  | |  | | |  | |  | | | |  | | | |  | | | |  | | | |  | | | |  | | | |  | | | |  |  |
| Self-rating of health | 0.17 | 0.09, 0.25 | | 0.00 | | |  | | 0.22 | | | | 0.14, 0.31 | | | | 0.00 | | | |  | | | | 0.73 | | | | 0.59, 0.86 | | | | 0.00 | | | |  |  |
| Poverty status (ref: No) |  |  | |  | | |  | |  | | | |  | | | |  | | | |  | | | |  | | | |  | | | |  | | | |  |  |
| Yes | -0.10 | -0.28, 0.09 | | 0.31 | | |  | | 0.67 | | | | 0.47, 0.88 | | | | 0.00 | | | |  | | | | -0.10 | | | | -0.40, 0.20 | | | | 0.51 | | | |  |  |
| Education (ref: high school degree/GED or less) | | | | | | |  |  | | | |  | | | |  | | | |  | | | |  | | | |  | | | |  | | | |  |  |  |
| Some college/Associates or more | 0.21 | 0.06, 0.35 | | 0.01 | | |  | | -0.17 | | | | -0.34, -0.01 | | | | 0.04 | | | |  | | | | 0.51 | | | | 0.27, 0.76 | | | | 0.00 | | | |  |  |
|  |  |  | |  | | |  | |  | | | |  | | | |  | | | |  | | | |  | | | |  | | | |  | | | |  |  |
| Primarily responsible for children <18-years-old | 0.04 | -0.16, 0.23 | | 0.71 | | |  | | 0.14 | | | | -0.08, 0.35 | | | | 0.21 | | | |  | | | | 0.01 | | | | -0.29, 0.32 | | | | 0.93 | | | |  |  |
| Caregiver employment (ref=not employed) |  |  | |  | | |  | |  | | | |  | | | |  | | | |  | | | |  | | | |  | | | |  | | | |  |  |
| Part/Full-time | 0.23 | 0.06, 0.40 | | 0.01 | | |  | | 0.18 | | | | 0.00, 0.36 | | | | 0.05 | | | |  | | | | 0.14 | | | | -0.13, 0.41 | | | | 0.30 | | | |  |  |
| Caregiver-patient gender concordance (ref=discordant) |  |  | |  | | |  | |  | | | |  | | | |  | | | |  | | | |  | | | |  | | | |  | | | |  |  |
| Aligned | 0.09 | -0.13, 0.30 | | 0.44 | | |  | | 0.02 | | | | -0.22, 0.25 | | | | 0.88 | | | |  | | | | 0.07 | | | | -0.29, 0.42 | | | | 0.72 | | | |  |  |
|  | Social/emotional burden^1^ | | | | | |  | | Financial burden^1^ | | | | | | | | | | | |  | | | | Health burden^2^ | | | | | | | | | | | |  |  |
|  | Beta | 95% CI | | P | | |  | | Beta | | | | 95% CI | | | | P | | | |  | | | | Beta | | | | 95% CI | | | | P | | | |  |  |
| Caregiver-patient relationship quality | -0.53 | -0.69, -0.36 | | 0.00 | | |  | | 0.02 | | | | -0.17, 0.20 | | | | 0.85 | | | |  | | | | -0.47 | | | | -0.71, -0.23 | | | | 0.00 | | | |  |  |
| ***Mediators*** |  |  | |  | | |  | |  | | | |  | | | |  | | | |  | | | |  | | | |  | | | |  | | | |  |  |
| Caregiver-patient communication quality (ref=Not well at all / A little well) | | | | | | | | | |  | | | |  | | | |  | | | |  | | | |  | | | |  | | | |  | | | |  |
| Somewhat well / Very well | -0.29 | -0.53, -0.05 | | 0.02 | | |  | | 0.00 | | | | -0.27, 0.26 | | | | 0.98 | | | |  | | | | 0.02 | | | | -0.34, 0.37 | | | | 0.93 | | | |  |  |
|  |  |  | |  | | |  | |  | | | |  | | | |  | | | |  | | | |  | | | |  | | | |  | | | |  |  |
| Social support | -0.32 | -0.40, -0.24 | | 0.00 | | |  | | -0.25 | | | | -0.34, -0.16 | | | | 0.00 | | | |  | | | | -0.30 | | | | -0.43, -0.17 | | | | 0.00 | | | |  |  |
|  |  |  | |  | | |  | |  | | | |  | | | |  | | | |  | | | |  | | | |  | | | |  | | | |  |  |
| Preparedness for caregiving | -0.36 | -0.46, -0.26 | | 0.00 | | |  | | -0.14 | | | | -0.25, -0.03 | | | | 0.01 | | | |  | | | | -0.31 | | | | -0.46, -0.15 | | | | 0.00 | | | |  |  |
| *p < 0.05, **p < 0.01, ***p < 0.001; Models estimated with imputed data | | | | | | | | | | | | | | | | | | | | | | | | | | | | | | | | | | | |  |  |  |
| ^1^Ordinary least squares regression model  ^2^Binary logit regression model | | | | | |  |  |  |  |  |  |  |  |  |  |  |  |  |  |  |  |  |  |  |  |  |  |  |  |  |  |  |  |  |  |  |  |  |
